# Differences in Virulence Factors and Antimicrobial Susceptibility of Uropathogenic *Enterococcus* spp. Strains in a Rural Area of Uganda and a Spanish Secondary Hospital

**DOI:** 10.3390/tropicalmed8050282

**Published:** 2023-05-16

**Authors:** Félix Carrasco Calzada, John Jairo Aguilera, Jaime Esteban Moreno, Juan Cuadros González, David Roca Biosca, Laura Prieto-Pérez, Ramón Pérez-Tanoira

**Affiliations:** 1 Clinical Microbiology Department, Hospital Universitario Príncipe de Asturias, 28805 Alcalá de Henares, Spain; felix.carrasco@edu.uah.es (F.C.C.); juan.cuadros@uah.es (J.C.G.); 2 Health Sciences Department, Faculty of Med, Universidad de Alcalá, 28805 Alcalá de Henares, Spain; 3 IIS-Fundación Jiménez Díaz, 28007 Madrid, Spain; jesteban@fjd.es (J.E.M.); lprietope@fjd.es (L.P.-P.); 4 CIBERINFEC-CIBER de Enfermedades Infecciosas, Instituto de Salud Carlos III, 28222 Madrid, Spain; 5 Máster Medicina Tropical y Salud Internacional, Universidad Autónoma de Madrid, 28049 Madrid, Spain; davidrocabiosca@yahoo.es; 6 Fundación El Alto, 12500 Vinaroz, Spain

**Keywords:** urinary tract infections, *Enterococcus* spp., virulence factors, antimicrobial susceptibility

## Abstract

*Enterococcus faecalis* and *Enterococcus faecium* have become two of the most important agents of nosocomial diseases due to their constantly growing resistance. Enterococcal infections are associated with biofilms, which are intrinsically sensitive to antimicrobials. The main goal of this study was to compare and relate their capacity to form biofilm and their antimicrobial sensitivity, as well as their virulence factors and their implicated genes, of strains isolated from patients with urinary tract infection (UTI) in a rural hospital in Uganda and a secondary hospital in Spain. A prospective study was conducted with 104 strains of *E. faecalis* and *E. faecium* isolated from patients with suspected UTI and who presented leukocyturia at the Saint Joseph Kitgum hospital (Uganda) and at the Hospital Universitario Principe de Asturias (Spain). All microorganisms were identified in Spain by MALDI-TOF mass spectrometry. Antimicrobial susceptibility studies were carried out using the Vitek^®^ 2 system (Biomériux, France). The biofilm formation capacity was studied by photospectrometry. Phenotypic and genotypic virulence factors were studied in all cases by PCR or expression techniques. In Uganda, we found a higher incidence of *E. faecium* (65.3%, *n* = 32), contrary to the situation found in Spain where most of the bacteria found belonged to *E. faecalis* (92.7%, *n* = 51). All *E. faecalis* strains were found to have very low levels of resistance to ampicillin, imipenem, and nitrofurantoin. However, *E. faecium* exhibited more than 25% resistance to these antibiotics. Although the *esp* gene has been shown in the results obtained to be an important initial agent in biofilm formation, we have also demonstrated in this study the intervention of other genes when *esp* is not present, such as the *ace1* gene. No statistically significant relationships were found between the presence of *agg* and *gelE* genes and increased biofilm formation. The significant difference between the incidence of *E. faecalis* and *E. faecium* and biofilm formation, between samples from Spain and Uganda, shows us very different profiles between countries.

## 1. Introduction

Urinary tract infections (UTIs) are one of the most important causes of morbidity and health expenditure worldwide [1,2].

The most common etiologic agent in UTIs in Western countries is *Escherichia coli*, which is responsible for almost 70% of cases in Europe and the U.S.A. [3]. However, the etiology changes in developing countries, where we find a higher incidence of other species, such as *Enterococcus*.

Enterococci (specially *E. faecalis* and *E. faecium*), during the last 30 years, have become one of the most important etiologic agents in UTIs, endocarditis, and nosocomial infections (catheterized patients) [4,5]. Among their virulence factors, one of the most relevant is their high intrinsic antibiotic resistance and their ability to produce biofilm, even in adverse conditions [6].

### 1.1. Biofilm Production Mechanism

A biofilm is defined as a microbiological community characterized by cells irreversibly attached to a substrate, to other bacteria, or embedded in a matrix of extracellular polymeric substances (EPSs) produced by the bacteria themselves [7].

The mechanisms of biofilm formation are well known for many pathogenic bacteria, such as *Pseudomonas aeruginosa* and *Bacillus subtilis* [8,9]. However, in the case of Enterococci, these factors are poorly defined at present, although we do know that there are many determinants and mediators of the process [10,11].

During the initial adhesion phase, the first step in biofilm formation, a large number of surface adhesins, proteases, and glycolipids play an important role in biofilm development. *Enterococcus faecalis* virulence requires cell wall-associated proteins, including the sortase-assembled endocarditis and biofilm-associated pilus (Ebp), important for biofilm formation in vitro and in vivo [12]. This was detected in UTI, catheter-associated UTI (CAUTI), and infective endocarditis models, in which the depletion of the *ebp* gene dramatically decreased tissue colonization [13,14].

Likewise, the absence of surface adhesins such as aggregation substance (Agg), enterococcal surface protein (Esp), and adhesin to collagen I (AceI) drastically reduces adherence in in vivo models of human cells and decreases the ability to form biofilm [15,16]. The deletion of AceI in in vivo rat models of UTI and endocarditis, as well as *agg*, reduces the adherence to renal tissue epithelial cells [15,16,17,18], and *agg* reduces the adherence to renal tissue epithelial cells [19].

Biofilm formation is determined by colony population density signals [12]. Matrix modifications are regulated by the QS regulator FsrA, which modulates the expression of the *gelE* gene. This, in turn, encodes a protease and its deletion reduces bacterial expression in in vivo and in vitro models [20,21].

### 1.2. Antibiotic Resistance due to Biofilm Formation

One of the most important factors of these biofilm structures is their resistance to antimicrobial agents. Bacteria forming a mature biofilm can be up to 1000 times more resistant to some antibiotics than their planktonic counterparts [7]. Among other factors, this resistance may be due to a decreased diffusion through the matrix. Sometimes, as in the case of aminoglycosides, the rate of penetration into the biofilm is slower than the half-life of the antibiotic, or even the treatment time itself [22]. They are also favored by the increased transmission of resistance genes, changes in pH that can inactivate the antibiotic, expression of expulsion pumps, or the presence of metabolically inactive cells, which allow them to tolerate situations of extracellular stress by inactivating the sites of antibiotic action [23].

The aim of our research is to perform a comparative study between enterococci causing UTI in a tertiary hospital in northern Uganda and a tertiary hospital in Spain. For this purpose, we will compare the results obtained related to antibiotic resistance, virulence factors (phenotypic and genotypic), and biofilm formation capacity.

## 2. Materials and Methods

Sample collection was performed between April and May 2019 at Saint Joseph Kitgum Hospital, a 350-bed tertiary hospital located in the northern region of Uganda. Samples from Spain were collected during February 2020 at the Hospital Príncipe de Asturias, in Alcalá de Henares (Madrid), where antimicrobial resistance testing was also performed.

### 2.1. Inclusion Criteria and Samples Collection

This prospective study included patients older than 16 years of age with leukocyturia and a concentration in urine of 125 leukocytes per mL as well as UTI-compatible symptoms, including suprapubic pain, burning sensation when urinating, incontinence, lower back pain, or patients in whom the presence of nitrites in urine was detected in routine urinalysis. We excluded all patients who had received empirical antibiotic treatment prior to the collection of the urine sample as well as all patients who underwent broad-spectrum antibiotic treatment for any condition within the 2 weeks before the collection of the sample.

Urine samples were collected and processed according to standardized procedures, following instructions from the laboratory clinicians. Bacterial growths of the genus *Enterococcus* obtained from the processing of the samples collected in Uganda were frozen and transported for subsequent identification and study in Spain, together with the growths of the same genus obtained in Spain. The samples were identified by matrix-assisted laser desorption/ionization time-of-flight (MALDI-TOF) spectrometry (Biomériux, Lyon, France).

### 2.2. Antibiotic Resistance Study

Enterococcal isolates from routine clinical specimens collected in both Spain and Uganda were tested using the AST-P592 card in a Vitek2 system (BioMérieux, Nürtingen, Germany) according to EUCAST criteria. The obtained results against ampicillin, ciprofloxacin, levofloxacin, imipenem, nitrofurantoin, erythromycin, trimethoprim/sulfamethoxazole, gentamicin, linezolid, quinupristin/dalfopristin, and vancomycin were collected.

### 2.3. Virulence Factors

A study of the main virulence factors related to biofilm formation in all enterococci strains isolated in this study was carried out. The protocols and methodologies used are described below.

#### 2.3.1. Phenotypic Virulence Factors

Hydrophobicity (H). Cell surface hydrophobicity was determined following the methodology developed by Stepien-Pysniak et al. [24]. Each strain was subcultured on Columbia Agar (OXOID, Hampshire, UK) supplemented with 5% defibrinated horse blood (Pro Animali Company, Wroclaw, Poland) at 37 °C for 24 h, and then resuspended in 5 mL of 0.9% NaCl to an optical density (OD600) of 1.0 (A_0_). Subsequently, 1.7 mL of xylene was added to 50 mL glass test tubes and the mixtures were vortexed for 90 s. After phase separation (15 min), the optical density of the aqueous phase (A) was measured again and compared to the organic phase. The percentage of cell surface hydrophobicity (%H) of the strain adhering to xylene was calculated using the equation %H = [(A_0_ − A)/A_0_] × 100. Strains with hydrophobicity equal to or higher than 50% were considered hydrophobic.

Gelatinase. To assess *gelE* gene expression, gelatinase production was evaluated by inoculating enterococci strains on TSA agar plus 3% gelatinase [24]. The appearance of a hazy halo around the growth after 24 h of incubation at 37 °C and subsequent cooling to 4 °C for 30 min was considered positive for gelatinase production. *E. faecalis* ATCC29212 was used as a positive control.

Hemolysis. The hemolytic capacity of the strains was assessed by inoculating them on Columbia agar with 5% defibrinated horse blood and subsequent incubation at 37 °C for 24 h under aerobic conditions. Partial hemolysis was considered to be that which provided a greenish color in the growth zones after incubation, and total hemolysis was the one which showed a transparent background. *E. faecalis* ATCC29212 was used as a positive control [24].

#### 2.3.2. Genotypic Virulence Factors

The genotypic virulence factors analyzed in this study were those most related to biofilm formation: *efaA*, *agg*, *gelE*, *esp*, *cpd*, and *Ace1*. Their presence in the collected strains was studied by PCR techniques.

DNA extraction from the strains was performed using the Fluorotype DNA extraction kit (Qiagen, Hilden, Germany) following the manufacturer’s instructions. Specific primers were used to amplify the sequences to be determined (Table 1).

In the case of *Ace1*, *gelE*, and *agg* genes, a protocol described by *Mannu* et al. [25] was followed. The reaction was performed in a total volume of 50 µL of which 5 were DNA, 5 of each primer (2.5 µL forwards and 2.5 µL reverse), 2 µL of magnesium chloride at 25 mM concentration, 5 µL of dream taq buffer (Thermofisher scientific, Waltham, MA, USA), 0.4 µL of dream taq (Thermofisher scientific, Waltham, MA, USA), 1 µL of dNTPs, and the rest double-distilled water. PCR conditions were denaturation at 94 °C for 30 s, annealing at 55 °C for 30 s, and elongation at 72 °C for 1 min for 30 cycles. In the case of the *esp* gene, the same process was performed, adding a first denaturation of 2 min at 95 °C. The annealing temperature for the *cpd* and *efaA* genes was 54 °C. The products of these PCRs were analyzed in 2% agarose gel electrophoresis in TAE (1×) and visualized under UV light.

### 2.4. Biofilm Formation

Biofilm formation was first studied using the technique described by Stepanovic et al. [28]. Briefly, bacteria were seeded in 0.9% NaCl saline solution to a concentration of 0.5 on the MacFarland scale, then diluted 1/100 in BHI medium plus 2% glucose. It was then transferred in triplicate to a multiwell plate with 200 µL and cultured at 37 °C and 5% CO_2_ for 24 h. After incubation, the culture medium was carefully aspirated, and the well was washed 3 times with 200 µL of 0.9% NaCl saline. The remaining biofilm was fixed with 200 µL of absolute methanol for 20 min and dried on a heat bed at 60 °C. The fixed biofilm was stained with 2% crystal violet for 15 min, and then excess stain was removed by washing twice with sterile double-distilled water. Finally, the dye was solubilized with 200 µL of absolute ethanol and left to solubilize for 5 min before interpretation by spectrophotometry at 570 nm.

To interpret these results, the optical density (OD) of the controls was calculated and the OD of each strain was measured individually. The cut-off optical density (Odc) was then defined as three standard deviations (SDs) above the mean OD of the controls: Odc = mean OD of the controls + (3 × SD of the controls). The final OD of each strain is expressed as the OD value measured for each strain minus the Odc value (OD = OD strain-Odc).

For the global study of their virulence capacity, a representation was made using binary values (1 present, 0 absent), in order to obtain a representative value of the virulence of the strains according to their origin.

For the comparison of virulence factors related to biofilm formation of the strains obtained in Uganda with those obtained in Spain, a value of “1” was assigned to the presence or expression of the genes studied, and a value of “0” to their absence. The same analysis was carried out for the resistance to the antibiotics studied, giving a value of “1” when they were resistant and a value of “0” if they were sensitive.

### 2.5. Statistical Analysis

The statistical package IBM SPSS for Windows Version 22.0 (IBM Corp., Armonk, NY, USA) was used. Continuous variables are expressed as medians and interquartile ranges, while qualitative variables appear as absolute and relative frequencies. All data were evaluated using a one-sided unpaired Wilcoxon nonparametric test to compare two groups. Statistical significance was set at *p*-values ≤ 0.05.

## 3. Results

### 3.1. Incidence by Specie in Uganda and Spain

In Uganda, we found a higher incidence of *E. faecium* (65.3%, *n* = 32) compared to *E. faecalis*. Most of the bacteria found in Spain belonged to *E. faecalis* (92.7%, *n* = 51).

*E. faecium* strains isolated in Spain exhibited higher resistance values to most of the antibiotics tested: ampicillin (100%), ciprofloxacin (75%), imipenem (100%), gentamicin (25%), Quinupristin/Dalfopristin (50%), and linezolid (50%) (Table 2).

### 3.2. Virulence Factors

**Figure 1:**
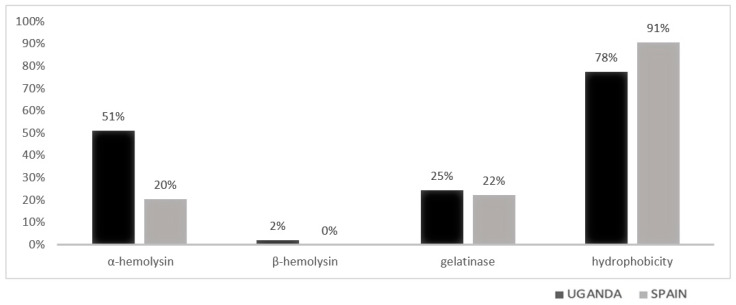
Phenotypic virulence factors isolated in each country expressed in percentage.

**Figure 2:**
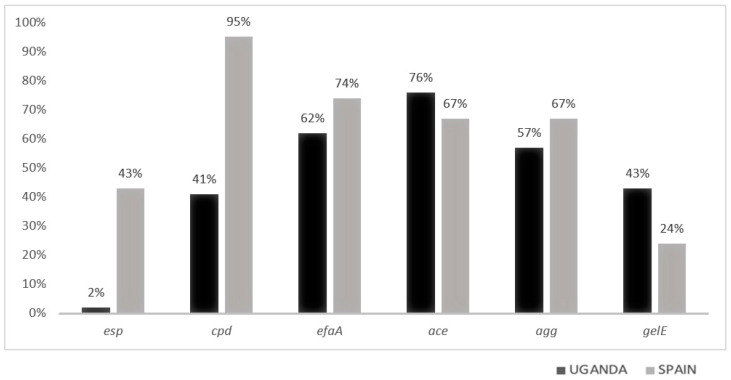
Genotypic virulence factors detected in strains isolated in Uganda and Spain.

Figure 1 (phenotypic factors) and Figure 2 (genotypic factors) show the distribution by place of origin in percentages of each of the virulence factors studied.

Among the 49 strains from Uganda, 51% had α-type hemolysis and 76% had the *Ace1* gene. The *esp* gene presence was practically absent. Although the *gelE* gene was found in more than 40% of the samples from the African country, it was only expressed in 22% of them (gelatinase).

A total of 54 enterococci were isolated in Spain, with the *cpd* gene present in most of the isolates (92%) and 41% of them had the *esp* gene. A total of 20% of these strains exhibited some type of hemolysis. The *gelE* gene was present in 22% of the isolates, but its expression was much higher in proportion to that obtained in Uganda (12 out of 13).

### 3.3. Biofilm Formation

Strains collected in Uganda exhibit less biofilm formation with a median and IQR of 2.96 (2.06–4.37), compared to those isolated in Spain with 5.67 (2.84–14.24). Table 3 shows the results of the comparation between Uganda and Spain in the presence or absence of each virulence mechanism.

In the case of Uganda, the median of the sums in each strain for the virulence factors was 4, with a IQR equal to 3–5. In Spain, these values were 5 (4–6).

For antibiotic resistance, strains from Uganda had values of 3 (3–5) and those from Spain had values of 2 (2–4).

The analysis of the probability of a greater presence of virulence factors related to biofilm formation (Mann–Whitney test) as a function of origin offers a *p*-value ≤ 0.001, that is, the fact of presenting a greater capacity for biofilm formation is directly related to the place of origin of the sample.

The results obtained with the same statistical study but referring to the presence of antibiotic resistance as a function of the origin of the strains show a *p*-value equal to 0.011, i.e., the origin of the sample is also related to the fact of presenting higher or lower levels of antibiotic resistance.

## 4. Discussion

Biofilm formation is one of the main mechanisms for bacteria to adapt to their environment. In the case of the genus *Enterococcus*, it allows them to adopt a high resistance to hostile environments with variations in pH and sudden changes in temperature [22,23].

The capacity for biofilm formation in *E. faecalis* and *E. faecium* species has been extensively studied in recent years, and several studies have compared the genes involved, the importance of their development as an antimicrobial resistance mechanism, and the genetic differences in relation to biofilm formation between clinically important and commensal strains [12,29,30,31,32]. However, to the best of our knowledge, this is the first study on biofilm formation by clinical strains of enterococci, and their related genes, on the African continent.

The results obtained for *Enterococcus* strains isolated from UTI infections diagnosed in a rural hospital in northern Uganda have been compared with similar strains from patients diagnosed in a secondary hospital in Spain.

### 4.1. Antibiotic Resistance

The levels of antibiotic resistance detected in this study show similar values between *E. faecalis* strains from Spain and Uganda. All strains of this species were found to be fully sensitive or with very low levels of resistance to ampicillin, imipenem, linezolid, and nitrofurantoin. However, a 100% resistance to quinupristin/dalfopristin and about 30% of resistance to ciprofloxacin and gentamicin were found. This can be explained by the intrinsic resistance of *E. faecalis* to streptogramin A. The combination with resistance to streptogramin B present in some *E. faecium* subspecies justifies intermediate sensitivities to the quinupristin/dalfopristin combination, depending on whether one or both resistance genes are present [33]. Only one of the *E. faecalis* strains exhibited resistance to vancomycin.

The high sensitivity to Imipenem, linezolid, and nitrofurantoin is consistent with other studies of antimicrobial profiles in enterococci in countries such as Uganda [34], Iran [35], India [36], and South Africa [37]. The study performed by Solorzano et al. also shows a high sensitivity to nitrofurantoin [34]. Nitrofurantoin can be considered a suitable alternative in a resource-poor countries such as Uganda, given its affordability and availability [34]. Imipenem and linezolid are expensive antibiotics that are difficult to access in these countries, although some studies suggest that linezolid may be a good alternative to prevent reinfection with Gram-positive bacteria due to its good elimination in urine [38,39].

Ciprofloxacin resistance values are slightly higher in samples found in Uganda than in those from Spain, although they coincide with those obtained in other similar studies carried out between Chile and Spain [38]. Sensitivity to ampicillin in *E. faecalis* is 100% in both countries, in agreement with other recent studies [40,41].

On the other hand, resistance to gentamicin in strains from Uganda and imipenem in strains from Spain are statistically associated with biofilm formation. Recent studies link sub-inhibitory concentrations of antibiotics with increased biofilm formation in Enterococci [42] and, in the specific case of gentamycin, increased expression of genes related to biofilm formation, such as *efaA* [42,43]. We have found differences between the antibiotic resistance profile obtained for *E. faecium* in Uganda and that obtained in Spain, such as high resistance to ampicillin and Imipenem in the Spanish strains, but the small number of *E. faecium* isolated in Spain makes these results not statistically relevant.

From the results obtained from the Ugandan *E. faecium* isolates, the high resistance to imipenem is of concern, as it is an antibiotic that is not included in any therapeutic guide in this country. This could be explained by the general resistance of some *E. faecium* strains to betalactams due to the production of amino acid substitution for penicillin-binding proteins (PBPs) [44].

### 4.2. Virulence Factors

#### 4.2.1. Phenotypic Virulence Factors

The percentage of hydrophobic enterococci strains was very high in both countries, with 78% of strains in Uganda and 91% in Spain. Hydrophobicity is a very important factor in the ability of bacteria to form biofilms [24]. Its expression seems to increase in stressful situations, increasing biofilm production [45]. Ugandan *Enterococcus* strains exhibited alpha-type hemolytic capacity in 51% of cases, while Spanish strains did so in 20%. Biofilm formation in Uganda (2.96 (2.06–4.37)) was much lower than that obtained in Spain (5.67 (2.84–14.24)). These data can be related to the results obtained in a study conducted in China in 2018, which shows how the expression of cytolysins corresponds to weak biofilm formation [46,47].

Although gelatinase expression values were rather similar between strains from both origins (25% in Uganda and 22% in Spain), this was not the case for the presence of the *gelE* gene. The strains collected in Uganda presented a prevalence of the gene of 43% while the strains from Spain only 24%. The expression of the gene in Spain among strains with the gene was around 100%, while Ugandan strains expressed the gene in only one out of two carriers. Several recent studies have shown that the presence and expression of this gene is closely related to biofilm formation [48,49]. However, it has also been investigated in recent years how the expression of this gene may be regulated by the *fsrA*, *fsrB,* and *fsrC* genes [50]. A study by Liu et al. in 2020 shows how the regulation of these *fsr* genes can be modulated as a function of the glucose concentrations in the medium, thus favoring biofilm formation in the absence of glucose [51].

#### 4.2.2. Genotypic Virulence Factors (Biofilm Formation-Related)

In our study, we found a statistical association between increased biofilm formation and the presence of the *esp* gene in agreement with other research studies [16,24,25,51,52,53]. However, the presence of *esp* was practically nil in African strains (2%), much lower than that observed in strains collected in Spain (43%). This low value contrasts with those obtained in strains studied in other countries, although the lack of genetic studies on enterococci in African countries makes the comparison difficult [45,54].

It is interesting to note that we found a relationship between the presence of the *aceI* gene and biofilm formation. This gene has a great affinity for type IV collagen, which is very present in cardiac tissue [18], as well as in urethral and bladder tissues [17]. In the absence of the *esp* gene, strains possessing the *aceI* gene generate a higher amount of biofilm (3.97 (2.49–5.50)) than those without (2.39 (2.01–3.59)) (*p* = 0.024). Despite this interest, to our knowledge, no one has studied this relationship and more samples need to be studied to confirm it, which could shed new light on the mechanisms of enterococcal biofilm formation.

The presence of the *cpd* gene was 95% in Spain and 41% in Uganda. These results are due to a higher incidence of *E. faecalis* species, which is in line with the results obtained in similar studies [50,55,56]. The presence of the *cpd* gene is associated with increased virulence and antibiotic resistance [52,55]. We found that biofilm formation increased when both *cpd* and *esp* were present (*p* < 0.001). This synergistic effect with *esp* was not found with the other genes studied. This result could indicate that, although esp is a very important factor in the first steps of biofilm formation, the *cpd* gene may interfere with the amount of biofilm once biofilm formation has started.

The *efaA* gene, or *E. faecalis* antigen A, is usually characteristic of strains causing UTIs [40]. It is of great importance in the adhesion phases of the biofilm to collagen and cardiac or renal tissue [57,58]. The results obtained in our study show the presence of this gene in 61.2% of the samples from Uganda and 74.1% of those from Spain. These results are close to those obtained in similar studies [40]. We obtained a statistically significant relationship between increased biofilm development and the presence of this gene only in the Ugandan strains.

In this study, no statistically significant relationship was found between the presence of the *agg* gene and the increase or decrease in biofilm production in contrast to other studies [47,54,58,59]. Another study from Egypt presents the possibility that the *agg* gene has a more important influence on the differentiation between moderate and strong biofilm formers, with its presence being a factor in the increase in biofilm formation [10].

### 4.3. Country Differences in Biofilm Formation

The results obtained when analyzing biofilm formation in the strains collected in Uganda show a median and IQR of 2.96 (2.06–4.37), while in Spain the result is 5.67 (2.84–14.24). Although there are no studies similar to this one on the African continent, the lower biofilm formation in Uganda could be due to different factors, such as the lower presence of the *esp* gene. Another possible factor that could affect these results is the stimulation of biofilm production by sub-inhibitory concentrations of certain antibiotics [59,60]. Continuous exposure to different antibiotics of these bacteria in Uganda and Spain could have been selected for agents with different genes in each country.

One of the most important limitations of this study is that neither whole genome sequencing nor single nucleotide polymorphism was available to identify bacterial strains, so we had to use MALDI-TOF (Bruker, Massachusetts, USA). However, whole genome sequencing is a more accurate and internationally used method to differentiate strains.

## 5. Conclusions

This is one of the first studies on virulence factors and antimicrobial susceptibility of *Enterococcus* strains in urinary tract infections in rural Uganda. The significant difference found when comparing the incidence of *E. faecalis* and *E. faecium* and biofilm formation between samples from Spain and Uganda shows us very different profiles between countries, whose in-depth study could allow us to better understand the virulence mechanisms of these bacteria.

Although the *esp* gene has been shown in the obtained results to be an important initial agent in biofilm formation, we have also demonstrated in this study the intervention of other genes when *esp* is not present, such as the *ace1* gene, which can initiate this process without the need for *esp*.

## Figures and Tables

**Table 1:** Nucleotide sequence of the primers used for each gene.

Virulence Genes	Nucleotide Sequence (5′-3′)	Size (pb)	Reference
*ace1*	AAAGTAGAATTAGATCCACAC	320	L. Mannu et al. [25]
	TCTATCACATTCGGTTGCG		
*agg*	AAGAAAAAGAAGTAGACCAAC	1553	Eaton et al. [26]
	AAACGGCAAGACAAGTAAATA		
*cpd*	TGGTGGGTTATTTTTCAATTC		Mannu et al. [25]
	TACGGCTCTGGCTTACTA		
*efaA*	GACAGACCCTCACGAATA	705	Eaton et al. [26]
	AGTTCATGCTGTAGTA		
*gelE*	ACGCATTGCTTTTCCATC	419	Eaton et al. [26]
	ACCCCGTATCATTGGTT		
*esp*	TTACCAAGATGGTTCTGTAGGCAC	913	Shankar et al. [27]
	CCAAGTATACTTAGCATCTTTTGG		

**Table 2:** Antibiotic resistance profile of strains isolated in Uganda and Spain.

Antibiotic	Species	Uganda (*E. faecalis n* = 18, *E. faecium n* = 32)	Spain (*E. faecalis n* = 51, *E. faecium n* = 4)
Ampicilin	*E. faecalis*	0% (0)	0% (0)
	*E. faecium*	31.2% (10)	100% (4)
Ciprofloxacin	*E. faecalis*	22.2% (4)	19.6% (10)
	*E. faecium*	28.1% (9)	75% (3)
Imipenem	*E. faecalis*	0% (0)	0% (0)
	*E. faecium*	31.2% (10)	100% (4)
Gentamycin	*E. faecalis*	27.8% (5)	27.4% (14)
	*E. faecium*	6.2% (2)	25% (3)
Vancomycin	*E. faecalis*	5.6% (1)	7.7% (3)
	*E. faecium*	0% (0)	50% (2)
Quinupristin/Dalfopristin	*E. faecalis*	100% (18)	100% (51)
	*E. faecium*	21.9% (7)	50% (2)
Linezolid	*E. faecalis*	5.6% (1)	0% (0)
	*E. faecium*	0% (0)	50% (2)
Nitrofurantoina	*E. faecalis*	0% (0)	3.8% (2)
	*E. faecium*	25% (8)	100% (4)

**Table 3:** Statistical relationship between the virulence factors studied versus their capacity for biofilm formation.

Virulence Mechanism		Uganda (*n* = 50)			Spain (*n* = 55)	
			Biofilm Formation. Median (IQR) (*n*)	*p*-Value	Biofilm Formation. Median (IQR) (*n*)	*p*-Value
**Phenotypics**	α-Hemolysis	+	2.36 (1.85–3.2) (25)	**0.015**	6.39 (1,64–28,76) (11)	0.817
		−	3.55 (2.60–4.51) (25)		5.58 (2.99–13.32) (43)	
	β Hemolysis	+	3 (1)	-	(0)	-
		−	2.95 (2.02–4.39) (49)		5.67 (2.84–14.24) (54)	
	Gelatinase	+	3.1 (2.33–3.91) (13)	0.182	3.98 (2.14–5.42) (12)	**0.042**
		−	2.83 (2.01–4.37) (37)		6.79 (3.54–23.04) (42)	
	hydrophobicity	+	2.66 (2.06–3.75) (39)	0.496	5.99 (3.12–14.42) (49)	**0.029**
		−	3.69 (2.13–4.54) (11)		1.64 (1.16–4.25) (5)	
**Genotypics**	esp	+	1.18 (1)	0.080	10.96 (5.01–29.41) (23)	**0.046**
		−	2.97 (2.15–4.40) (49)		5.06 (2.53–9.57) (31)	
	cpd	+	2.97 (2.41–4.32) (20)	0.276	5.99 (3.55–16.46) (51)	**≤** **0.001**
		−	2.71 (1.89–4.23) (30)		1.22 (1.08–1.50) (3)	
	efaA	+	3.63 (2.47–4.6) (30)	**0.004**	5.38 (2.64–11.16) (40)	0.317
		−	2.29 (1.80–3.10) (20)		6.39 (4.01–31.51) (14)	
	Ace1	+	3.24 (2.31–4.54) (38)	**0.030**	5.56 (2.99–14.15) (36)	0.958
		−	2.22 (1.68–2.44) (12)		5.67 (2.51–18.69) (18)	
	agg	+	2.98 (2.31–4.54) (29)	0.300	5.21 (2.72–12.1) (36)	0.272
		−	2.50 (1.95–3.81) (21)		6.79 (4.02–31.52) (18)	
	gelE	+	3.71 (2.43–4.44) (21)	0.079	5.26 (5.02–9.99) (13)	0.593
		−	2.44 (1.94–3.10) (29)		6.03 (2.68–26.77) (41)	

Bold results are the ones which are statistically significant.

## Data Availability

Not applicable.
